# Impact of high versus low fixed loads and non-linear training loads on muscle hypertrophy, strength and force development

**DOI:** 10.1186/s40064-016-2333-z

**Published:** 2016-05-20

**Authors:** Julius Fink, Naoki Kikuchi, Shou Yoshida, Kentaro Terada, Koichi Nakazato

**Affiliations:** Graduate Schools of Health and Sport Science, Nippon Sport Science University, 7-1-1, Fukasawa, Setagaya-ku, Tokyo, 158-8508 Japan; Department of Training Science, Nippon Sport Science University, 1221-1, Kamoshidacho, Aoba-ku, Yokohama, 227-0033 Japan

**Keywords:** Cross-sectional area, Maximum voluntary contraction, Periodized training

## Abstract

**Background:**

In this study, we investigated the effects of resistance training protocols with different loads on muscle hypertrophy and strength.

**Methods:**

Twenty-one participants were randomly assigned to 1 of 3 (n = 7 for each) resistance training (RT) protocols to failure: High load 80 % 1RM (8–12 repetitions) (H group), low load 30 % 1RM (30–40 repetitions) (L group) and a mixed RT protocol (M group) in which the participants switch from H to L every 2 weeks. RT consisted of three sets of unilateral preacher curls performed with the left arm 3 times/week with 90 s rest intervals between sets. The right arm served as control. Maximum voluntary contraction (MVC) of the elbow flexors (elbow angle: 90°) and rate of force development (RFD, 0–50, 50–100, 100–200 and 200–300 ms) were measured. Cross-sectional area (CSA) of the elbow flexors was measured via magnetic resonance imaging (MRI). All measurements were conducted before and after the 8 weeks of RT (72–96 h after the last RT). Statistical evaluations were performed with two-way repeated measures (time × group).

**Results:**

After 8 weeks of 3 weekly RT sessions, significant increases in the left elbow flexor CSA [H: 9.1 ± 6.4 % (p = 0.001), L: 9.4 ± 5.3 % (p = 0.001), M: 8.8 ± 7.9 % (p = 0.001)] have been observed in each group, without significant differences between groups. Significant changes in elbow flexor isometric MVC have been observed in the H group (26.5 ± 27.0 %, p = 0.028), while no significant changes have been observed in the M (11.8 ± 36.4 %, p = 0.26) and L (4.6 ± 23.9 %, p = 0.65) groups. RFD significantly increased during the 50–100 ms phase in the H group only (p = 0.049).

**Conclusions:**

We conclude that, as long as RT is conducted to failure, training load might not affect muscle hypertrophy in young men. Nevertheless, strength and RFD changes seem to be load-dependent. Furthermore, a non-linear RT protocol switching loads every 2 weeks might not lead to superior muscle hypertrophy nor strength gains in comparison with straight RT protocols.

## Background

Among several other RT parameters such as rest interval between sets, total volume, time under tension and concentric vs. eccentric training, the optimal training load for muscle and strength gains has been widely investigated in previous research (Buresh et al. 2009; Campos et al. 2002; American College of Sports 2009; Ogasawara et al. 2013; Schuenke et al. 2012; Wilborn et al. 2009; Kraemer et al. 1990). The general opinion is that heavy load is necessary to stimulate fast twitch muscle fibers with the greatest potential for hypertrophy (Fry 2004). Although previous research showed that a training load of 60–90 % 1RM maximizes muscle protein synthesis (MPS) (Kumar et al. 2009), the optimal training load for muscle gains is inconclusive, especially if training is performed to volitional failure (Sampson and Groeller 2015; Schoenfeld et al. 2015).

Recent research has shown that low load RT leads to similar muscle hypertrophy gains compared to high load RT (Ogasawara et al. 2013; Schoenfeld et al. 2015; Mitchell et al. 2012). Indeed, Mitchell et al. (2012) showed that 30 % 1RM induces comparable muscle gains when compared to 80 % 1RM, both conditions performed to failure by recreationally active participants with no former weightlifting experience. Similar results have been observed in a study comparing the MPS rate after a bout of RT comparing 30 % 1RM and 90 % 1RM to failure (Burd et al. 2010). Another recent study investigating the effects of different loads on muscle cross-sectional area (CSA), strength and endurance changes in a well trained cohort demonstrated similar results with regard to muscle hypertrophy after a period of 8 weeks for the 25–35 repetitions group and the 8–12 repetitions group, both groups training to volitional failure (Schoenfeld et al. 2015).

Strength and power improvements are also important factors to consider when selecting a RT protocol. Indeed, muscle hypertrophy may not be directly correlated with strength increases (Sale 1988). Despite similar muscle mass gains in both low and high load RT, strength increases have been observed in high load RT, while improved endurance has been recorded in low load RT (Schoenfeld et al. 2015). Therefore, high load RT might lead to superior results in strength as compared to low load RT (Schoenfeld et al. 2015; Ogasawara et al. 2013). Similar to strength gains, the rate of force development (RFD) responses to different training loads has been widely examined for practical applications in sports (Haff and Nimphius 2012). Regardless of such practical importance, RFD responses to regular RT with fixed tempo have not been fully investigated so far.

To overcome suboptimal strength gains in low load RT, we hypothesized that a non-linear RT protocol mixing loads might be effective. A review including several studies switching loads every 2–3 weeks conducted with an intensity of 3—12RM 3 times/week for a period of 7 weeks (McGee et al. 1992; Stowers et al. 1983) showed that non-linear periodized RT can be superior to regular RT with regard to body fat, body weight, strength, power and endurance in experiments as short as 6 weeks (Fleck 1999). However, previous research on periodized RT protocols did not include very low load (<30 % 1RM) RT. Especially the effects of periodically switching loads on muscle hypertrophy have not been studied so far.

The aim of this study is to compare the effects of low, high and mixed load RT protocols with fixed tempo on chronic muscle hypertrophy, strength and RFD changes. We assumed that mixing very low (30 % 1RM) and high load (80 % 1RM) protocols may lead to superior muscle gains as compared to low or high load only protocols, because of different ranges of mechanical stimulations. Since strength gains have been shown to be load-dependent (Ogasawara et al. 2013; Schoenfeld et al. 2015), we hypothesized that effects on strength gains might be superior to low load RT, but inferior to high load RT in the non-linear RT protocol. Indeed, the low load phases might impair neuromuscular adaptations expected to occur with high load RT only (Gabriel et al. 2006). With regard to RFD responses, we examined if the load of a RT performed with fixed tempo affects the explosive power output. To the best of our knowledge, the effects with regard to RFD in non-linear RT protocols with controlled velocity have never been studied so far. Similar to strength adaptations, the different stimulations in non-linear RT might impair neuromuscular adaptations (Gabriel et al. 2006). Since RFD improvements are important in many sports, this information will be beneficial for athletes and coaches in selecting RT training loads.

## Methods

### Subjects

Twenty-one young male gymnastics athletes unaccustomed to resistance training volunteered to participate in this study. The participants did not refrain from their usual gymnastics training but refrained from doing any resistance training during the duration of the experiment. All the participants were informed about the potential risks of the experiment and gave their written consent to participate in the experiment. The sample size was calculated (GPower 3.1, Dusseldorf, Germany) a priori as follows: Effect size f = 0.25, αerr prob = 0.05, power = 0.8. The required total sample size was *n* = 21, n = 7 for each group. This study was approved by the Ethics Committee of the Nippon Sport Science University and was in accordance with the Declaration of Helsinki for Human Research.

### Resistance training

In order to get accurate results concerning hypertrophic gains in a specific muscle group, we chose unilateral biceps preacher curls because of their unique isolation and control ability. By locking the arm on the bench, swinging and involvement of different muscles can be avoided. The right arm was the dominant arm for all participants in this study. In accordance with previous research (Kawakami et al. 1995) and in order to minimize outside effects from other daily activities, training was performed with the left arm and the right arm serving as control. Moreover, a previous study demonstrated that indirect muscle damage markers are not significantly different between the dominant and non-dominant arms (Newton et al. 2013). Unilateral training was chosen in order to have the untrained arm as a control, since muscle hypertrophy can occur due to activities different than the prescribed RT protocol. Participants were randomly assigned to 1 of the 3 following groups: the H group (3 sets of 80 % 1RM), the L group (3 sets of 30 % 1RM), and the M group (training protocols changing every 2 weeks starting with 2 weeks at 80 % 1RM followed by 2 weeks at 30 % 1RM and so on). Rest intervals between sets were 90 s for all the groups. RT was conducted 3 times/week with the left arm. Every set was taken to volitional failure with a cadence of 1 s for the concentric and 2 s for the eccentric part of the movement.

Participants refrained from participating in any other RT training during the duration of the experiment and were familiarized with the exercise 2 weeks prior to the start of the experiment by qualified trainers.

RT sessions were supervised by qualified trainers in order to ensure correct execution of the exercises. If a trainee was able to complete more than 8 or 35 repetitions in the H and L groups respectively, the load was increased by 5 % for the following sessions.

### Measurements

#### Muscle CSA

Participants underwent magnetic resonance imaging (MRI) scans of both the trained and the non-trained control upper arms including the biceps, the brachialis and the triceps muscles during the week before training start and between 72 and 96 h after the last training session (week 9). To ensure accuracy of the measurements, markers filled with water were placed exactly at half-distance of each participant’s upper arm (measured from the elbow joint to the shoulder joint). Participants lay with their right arm in a supinated position. Beginning at the joint line, 20 axial scans were taken. The following parameters have been used to acquire images: repetition time/echo time, 460 m s/26 m s; field of view 20 cm, phase/frequency, 320; slice thickness, 3 mm; gap, 10 mm. The images showing the markers were then analyzed via ImageJ (National Institutes of Health) and the square area of each cut was calculated twice by the same investigator. The mean value of the 2 measurements was used for calculations. A reliability test showed an intraclass correlation coefficient (ICC) of >0.9 for our CSA calculations.

#### Muscle strength and RFD

Maximum voluntary contraction (MVC) has been measured during the week before training start and between 72 and 96 h after the last training session by using Biodex system 3 (Biodex Medical systems, Inc., USA). MVC measurements were performed after the MRI measurement (week 9). After one warm-up set (20–30 % 1RM) of barbell curls, the participants were seated on a chair and the left arm was strapped to an horizontal support at chest height, so that the elbow joint was at the same height as the handle joint (shoulder supination angle 90°). The participants were holding the Biodex handle in an elbow supination position at 90° (0° at full extension). Each participant performed 2 MVC’s (contraction time: 5 s) separated by 60 s rest intervals. Before each measurement, the participants were instructed to pull the handle parallel to the ground with maximal force. The highest value was recorded for each participant. ICC was >0.9 for MVC measurements.

RFD (Nm/s) was calculated from onset of contraction when the arm flexor torque exceeded baseline by 7.5 Nm (Aagaard et al. 2002) with a sampling rate of 100 Hz. Relative RFD was calculated by dividing RFD by MVC (%MVC/s). ICC for RFD was >0.9.

### Statistical analyses

Data are shown as mean ± SD. We used two-way analysis of variance (ANOVA) (time × groups) to analyze the significance of our values and post hoc Bonferroni tests (SPSS for Macintosh version 22.) when appropriate. ICC was calculated via a reliability test for each measurement. The significance level was set at p < 0.05. We also calculated the effect size (ES) (Cohen 1988) for each group and parameter. According to Cohen, ES = 0.2 is considered to be a ‘small’ effect size. ES = 0.5 represents a ‘medium’ effect size. ES = 0.8 means a ‘large’ effect size.

## Results

### Participant characteristics

Average age, body mass, height and body fat for each group are shown in Table 1. No significant differences in any of the parameters among groups were observed.

**Table 1:** Participant characteristics

Group	Age (years)	Body mass (kg)	Height (cm)	Body fat (%)
H	23.4 ± 3.0	64.6 ± 4.9	167.5 ± 2.1	12.1 ± 4.4
M	23 ± 3.1	62.3 ± 4.0	167.5 ± 3.7	11.5 ± 4.3
L	23.1 ± 2.4	63.2 ± 5.6	170.7 ± 6.4	12.0 ± 3.2

All values are mean ± SD. H: high load (80 % 1RM), L: low load (30 % 1RM), M: mixed (switch between 80 and 30 % 1RM every 2 weeks)

### Muscle CSA changes

**Fig. 1:**
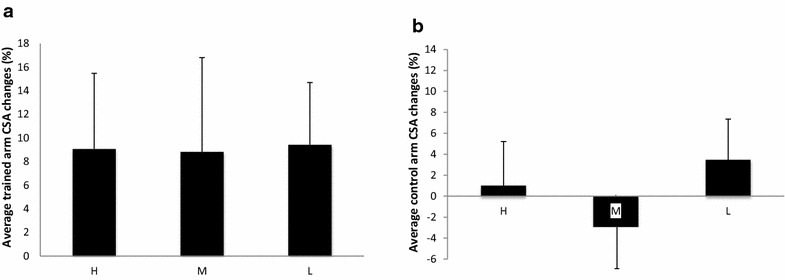
CSA changes after 8 weeks of strength training. Average CSA changes (%) (±SD) after 8 weeks in the trained (**a**) and untrained (**b**) arm. H: high load (80 % 1RM), L: low load (30 % 1RM), M: mixed (switch between 80 and 30 % 1RM every 2 weeks). *p < 0.05 versus before

There was no significant difference for the initial CSA values among groups (Table 2). Two-way ANOVA analysis showed main effects (time) for each group (F = 45.4, p < 0.001). The L group’s trained arm CSA changed 9.4 ± 5.3 % (p = 0.001) as compared to 9.1 ± 6.4 % (p = 0.001) for the H group and 8.8 ± 7.9 % (p = 0.001) for the M group. No significant differences of CSA changes between groups were observed. The right control arm did not significantly change in any of the groups (Fig. 1).

**Table 2:** CSA and MVC changes

Group	CSA (cm^2^) pre	CSA (cm^2^) post	ES	MVC (Nm) pre	MVC (Nm) post	ES
H	9.7 ± 1.6	10.6 ± 1.5*	0.6	61.5 ± 6.5	77.8 ± 21.0*	1.1
M	10.3 ± 1.8	11.2 ± 1.9*	0.5	67.4 ± 15.0	75.3 ± 21.0	0.4
L	9.7 ± 1.1	10.7 ± 0.9*	0.9	68.4 ± 23.5	71.5 ± 15.3	0.15

All values are mean ± SD. H: high load (80 % 1RM), L: low load (30 % 1RM), M: mixed (switch between 80 % and 30 % 1RM every 2 weeks)

*CSA* cross-sectional area, *MVC* maximum voluntary contraction, *RFD* rate of force development, *ES* effect size

* p < 0.05 versus pre

### Muscle strength and RFD

**Fig. 2:**
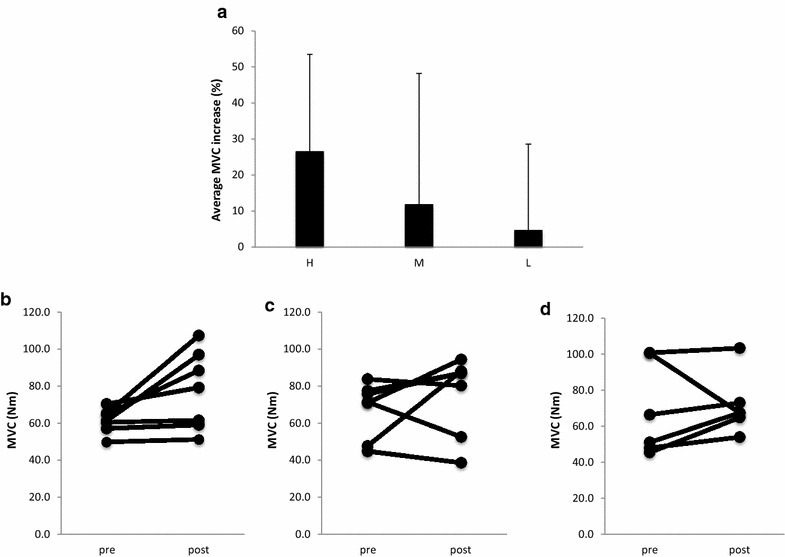
MVC changes after 8 weeks of strength training. Average MVC changes (%) (±SD) after 8 weeks in the trained arm (**a**). Individual MVC changes before and after 8 weeks in the trained arm (**b** high load (H, 80 % 1RM), **c** mixed (M, switch between 80 and 30 % 1RM every 2 weeks), **d** low load (L, 30 % 1RM). *p < 0.05 versus before

There were no significant differences for the initial MVC and RFD values among groups (Table 2). Two-way ANOVA analysis showed main effects (time) for strength (F = 5.4, p = 0.032). The H group increased strength 26.5 ± 27.0 % (p = 0.028), while no significant changes could be observed in the M (11.8 ± 36.4 %, p = 0.26) and L (4.6 ± 23.9 %, p = 0.65) groups (Fig. 2).

**Fig. 3:**
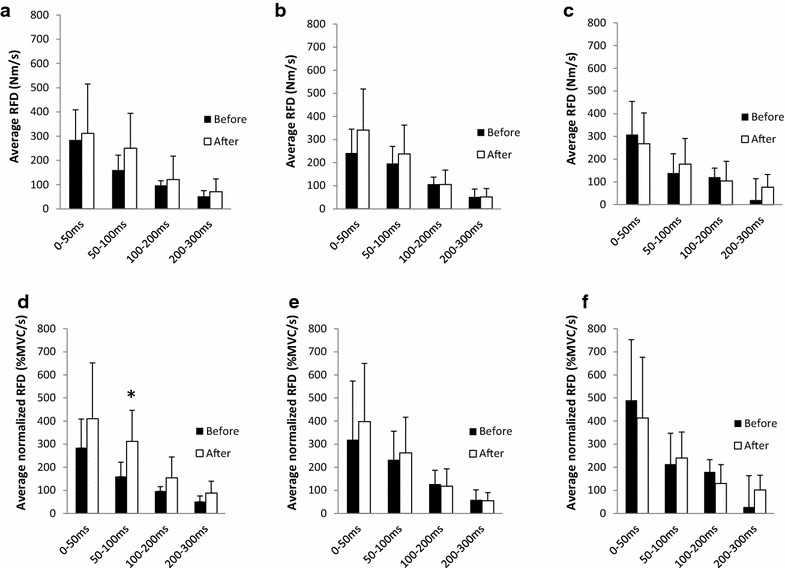
RFD changes after 8 weeks of strength training. Average RFD (±SD) before and after the training period for the H (**a**), M (**b**) and L (**c**) groups. Average relative RFD (%MVC/s) (±SD) before and after the training period in the early phase for the H (**d**), M (**e**) and L (**f**) groups. H: high load (80 % 1RM), L: low load (30 % 1RM), M: mixed (switch between 80 and 30 % 1RM every 2 weeks). *p < 0.05 versus before

Two-way ANOVA analysis showed main effects (time) for RFD in the 50–100 ms phase of normalized RFD (F = 4.5, p = 0.049). The H group increased 95.6 ± 310.6 %, while the L and M groups did not show any significant changes. No significant differences inside groups could be observed (Fig. 3).

### Number of repetitions

The average number of total repetitions was 15.3 ± 1.6 reps (1st set: 7.6 ± 0.7 reps, 2nd set: 4.6 ± 0.8 reps, 3rd set: 2.9 ± 1.1 reps) for the H protocol and 75.3 ± 12.6 reps (1st set: 38.3 ± 4.3 reps, 2nd set: 24.3 ± 6.6 reps, 3rd set: 12.7 ± 3.2 reps) in the L group.

## Discussion

To our knowledge, this is the first study directly comparing fixed load RT protocols including very low (30 % 1RM) and high load (80 % 1RM) training to a non-linear RT protocol. In this study, we investigated the hypothesis according to which a RT protocol with mixed loads leads to superior muscle gains in comparison with continuous RT protocols with high or low loads. Although each group showed a significant CSA increase in the trained arm, no differences among groups were observed. With regard to muscular strength, only the H group demonstrated significant increases. Furthermore, a significant RFD increase was observed in the 50–100 ms phase for the H group only.

The CSA increases of recreationally active individuals after both high and low load RT protocols in this study (H: 9.1 %, L: 9.4 %) are in line with previous research, showing that low load and high load RT protocols lead to similar muscle gains if every set is conducted to failure (Schoenfeld et al. 2015; Ogasawara et al. 2013; Mitchell et al. 2012; Weiss et al. 2000). Indeed, type I and II fiber area increases were observed in previous studies regardless of training load (30 vs. 80 % 1RM) with no differences between groups (Mitchell et al. 2012). Previous research (Fry 2004) showed that, if each set is conducted to failure, the effort is the same leading to similar muscle fiber activation no matter the training load. Thus, muscle hypertrophy seems to be independent from the training load as long as effort is the same (Burd et al. 2012; Mitchell et al. 2012). It should be noted that, in general, type I fibers are not larger than type II fibers, especially in young men (Staron et al. 2000). Thus, even if type I fibers are hypertrophied, the same amount of muscle hypertrophy might not be reached. Although our measurement schedule was set 72–96 h after the last RT (Schoenfeld et al. 2015; Ogasawara et al. 2013) in order to avoid increased CSA results due to acute muscle swelling, we cannot exclude the possibility of minor influences of remaining muscle swelling on our CSA results. Indeed, a certain amount of CSA increases observed in short term studies (~3–4 weeks of RT) may be partly due to muscle edema and not to pure muscle hypertrophy (Damas et al. 2016).

Even though nonlinear periodized high load RT has shown benefits with regard to body fat, body weight, strength, power and endurance (Fleck 1999), our results demonstrated that a non-linear periodized RT protocol including low load RT bouts does not lead to superior CSA increases. Indeed, it has been shown previously that powerlifters mainly training at intensities >90 % 1RM preferentially increase type II muscle fibers as compared to bodybuilders training with moderate load and displaying equal hypertrophy in both fiber types (Fry 2004). However, each group performed RT to failure and probably activated a similar range of muscle fibers (Mitchell et al. 2012; Campos et al. 2002). As observed in previous studies, RT not performed to failure might not maximize muscle fiber activation, especially type II ibers may not be fully recruited (Taaffe et al. 1996). Although we confirmed that a straight, very low load RT with failure induced significant muscle hypertrophy, very low load RT did not induce additive hypertrophy in the nonlinear periodized RT.

We could observe a significant strength increase in the H group (26.5 %), similar to previous results observed in studies conducted on the upper body with high load RT (13.9–19.6 %) (Ogasawara et al. 2013; Schoenfeld et al. 2015). Even though not being significant, the L group demonstrated a small increase (4.6 %) in strength, in line with results observed in the same previous studies (2–8.8 %) with low load RT (Ogasawara et al. 2013; Schoenfeld et al. 2015). Even though not being significant, the strength increase (11.8 %) observed in the group mixing high and low load RT was between low (4.6 %) and high load (26.5 %) strength increases with a large effect size (0.4). Previous studies had shown superior strength gains for periodized training, however the periodization was within high load RT (2–10 RM) (Stone et al. 1981; Stowers et al. 1983). We propose that neuromuscular adaptations such as a greater neural output from the central nervous system in response to high RT might have been hindered by the low load RT bouts (Gabriel et al. 2006). Nevertheless, due to the small sample size in our study, individual external factors might have strongly affected our results. Indeed, in both the H and M groups, four subjects increased MVC while three subjects did not. However, only in the M group, one subject showed a strong decrease in MVC. The decrease of this single subject might have affected the outcome; therefore we cannot completely be sure if the results have been solely due to the training intervention or if external factors have influenced the results.

We also evaluated RFD for all groups. We found that high load RT with controlled tempo showed RFD increases in the early phase. Studies including RFD changes after fixed tempo RT are limited. Andersen et al. (Andersen et al. 2010) reported that high load (6–12RM) RT with controlled movement leads to late phase (>200 ms) RFD increase after 14 weeks of RT. Although we failed to observe statistical significance, high averaged values for normalized RFD were observed after RT in the H group. We suspect that a training period of 8 weeks is too short to obtain significant differences. On the other hand, low load and non-linear RT with fixed tempo seems to be suboptimal for improving RFD. Taken together, these results indicate that the different phases of RFD can significantly vary, depending on RT parameters such as total training period, speed of contraction and training load (Oliveira et al. 2013).

This study has been conducted with several limitations: First, concerning the total training volume (sets × load × repetitions), the L group showed a 1.5 times higher volume in comparison to the H group. However, we did not equate the total volume on purpose, in order to be consistent with previous research in the same field. Indeed, in numerous previous studies demonstrating similar results in muscle hypertrophy between low and high load RT, the total training volume was not equated (Schoenfeld et al. 2015; Mitchell et al. 2012; Ogasawara et al. 2013; Popov et al. 2006). Second, in our study we did not assess endurance, but a previous study showed improvements in the number of repetitions of 50 % 1RM bench press in a low load protocol as compared to a high load protocol (Schoenfeld et al. 2015). Therefore, we could expect the L group to improve the most. It might have been of interest to measure endurance in order to assess if strength and endurance both improve in a mixed protocol. Third, dietary intake has not been monitored for the period of the experiment. However, the major part of the participants had similar daily activities including dietary habits. Fourth, our sampling rate of 100 Hz for RFD was low, better results could have been obtained with higher frequencies. Fifth, the progression during the training period has only been assessed pre and post intervention without collecting data during the study. Furthermore, since we could not control every daily activity of the participants, the effects of activities involving endurance after resistance training might also have affected our results (Kikuchi et al. 2015).

In conclusion, we demonstrated that there are no significant differences with respect to muscular hypertrophy for different training loads, if RT is conducted to failure for a period of 8 weeks. Moreover, switching between different ranges of mechanical stimulations did not improve muscle hypertrophy or strength over a period of 8 weeks.

## Abbreviations

ANOVA: analysis of variance
CSA: cross-sectional area
ES: effect size
H: high load
ICC: intraclass correlation coefficient
L: low load
M: mixed load
MPS: muscle protein synthesis
MVC: maximum voluntary contraction
RFD: rate of force development
RT: resistance training

## References

[CR1] Aagaard P, Simonsen EB, Andersen JL, Magnusson P, Dyhre-Poulsen P (2002). Increased rate of force development and neural drive of human skeletal muscle following resistance training. J Appl Physiol.

[CR2] American College of Sports (2009). American College of Sports Medicine position stand. Progression models in resistance training for healthy adults. Med Sci Sports Exerc.

[CR3] Andersen LL, Andersen JL, Zebis MK, Aagaard P (2010). Early and late rate of force development: differential adaptive responses to resistance training?. Scan J Med Sci Spor.

[CR4] Burd NA, West D, Staples AW, Atherton PJ, Baker JM, Moore DR, Holwerda AM, Parise G, Rennie MJ, Baker SK (2010). Low-load high volume resistance exercise stimulates muscle protein synthesis more than high-load low volume resistance exercise in young men. PLoS ONE.

[CR5] Burd NA, Mitchell CJ, Churchward-Venne TA, Phillips SM (2012). Bigger weights may not beget bigger muscles: evidence from acute muscle protein synthetic responses after resistance exercise. Appl Physiol Nutr Metab.

[CR6] Buresh R, Berg K, French J (2009). The effect of resistive exercise rest interval on hormonal response, strength, and hypertrophy with training. J Strength Cond Res.

[CR7] Campos GE, Luecke TJ, Wendeln HK, Toma K, Hagerman FC, Murray TF, Ragg KE, Ratamess NA, Kraemer WJ, Staron RS (2002). Muscular adaptations in response to three different resistance-training regimens: specificity of repetition maximum training zones. Eur J Appl Physiol.

[CR8] Cohen J (1988). Statistical power analyses for the behavioral sciences.

[CR9] Damas F, Phillips SM, Lixandrão ME, Vechin FC, Libardi CA, Roschel H, Ugrinowitsch C (2016). Early resistance training-induced increases in muscle cross-sectional area are concomitant with edema-induced muscle swelling. Eur J Appl Physiol.

[CR10] Fleck SJ (1999). Periodized strength training: a critical review. J Strength Cond Res.

[CR11] Fry AC (2004). The role of resistance exercise intensity on muscle fibre adaptations. Sports Med.

[CR12] Gabriel DA, Kamen G, Frost G (2006). Neural adaptations to resistive exercise. Sports Med.

[CR13] Haff GG, Nimphius S (2012). Training principles for power. Strength Cond J.

[CR14] Kawakami Y, Abe T, Kuno S-Y, Fukunaga T (1995). Training-induced changes in muscle architecture and specific tension. Eur J Appl Physiol Occup Physiol.

[CR15] Kikuchi N, Yoshida S, Nakazato K (2015). The effect of high-intensity interval cycling sprints subsequent to arm-curl exercise on upper-body muscle strength and hypertrophy: a pilot study. J Strength Cond Res.

[CR16] Kraemer WJ, Marchitelli L, Gordon SE, Harman E, Dziados JE, Mello R, Frykman P, McCurry D, Fleck SJ (1990). Hormonal and growth factor responses to heavy resistance exercise protocols. J Appl Physiol.

[CR17] Kumar V, Selby A, Rankin D, Patel R, Atherton P, Hildebrandt W, Williams J, Smith K, Seynnes O, Hiscock N (2009). Age related differences in the dose–response relationship of muscle protein synthesis to resistance exercise in young and old men. J Physiol.

[CR18] McGee D, Jessee TC, Stone MH, Blessing D (1992). Leg and hip endurance adaptations to three weight-training programs. Eur J Appl Physiol Occup Physiol.

[CR19] Mitchell CJ, Churchward-Venne TA, West DW, Burd NA, Breen L, Baker SK, Phillips SM (2012). Resistance exercise load does not determine training-mediated hypertrophic gains in young men. J Appl Physiol.

[CR20] Newton MJ, Sacco P, Chapman D, Nosaka K (2013). Do dominant and non-dominant arms respond similarly to maximal eccentric exercise of the elbow flexors?. J Sci Med Sport.

[CR21] Ogasawara R, Loenneke JP, Thiebaud RS, Abe T (2013). Low-load bench press training to fatigue results in muscle hypertrophy similar to high-load bench press training. Int J Clin Med.

[CR22] Oliveira FB, Oliveira AS, Rizatto GF, Denadai BS (2013). Resistance training for explosive and maximal strength: effects on early and late rate of force development. J Sports Sci Med.

[CR23] Popov DV, Swirkun DV, Netreba AI, Tarasova OS, Prostova AB, Larina IM, Borovik AS, Vinogradova OL (2006). Hormonal adaptation determines the increase in muscle mass and strength during low-intensity strength training without relaxation. Hum Physiol.

[CR24] Sale DG (1988). Neural adaptation to resistance training. Med Sci Sports Exerc.

[CR25] Sampson JA, Groeller H (2015). Is repetition failure critical for the development of muscle hypertrophy and strength?. Scan J Med Sci Sports.

[CR26] Schoenfeld BJ, Peterson MD, Ogborn D, Contreras B (2015). Sonmez GT (2015) Effects of low-versus high-load resistance training on muscle strength and hypertrophy in well-trained men. J Strength Cond Res.

[CR27] Schuenke MD, Herman JR, Gliders RM, Hagerman FC, Hikida RS, Rana SR, Ragg KE, Staron RS (2012). Early-phase muscular adaptations in response to slow-speed versus traditional resistance-training regimens. Eur J Appl Physiol.

[CR28] Staron RS, Hagerman FC, Hikida RS, Murray TF, Hostler DP, Crill MT, Toma K (2000). Fiber type composition of the vastus lateralis muscle of young men and women. J Histochem Cytochem.

[CR29] Stone MH, O’Bryant H, Garhammer J (1981). A hypothetical model for strength training. J Sports Med Phys Fitness.

[CR30] Stowers T, McMillan J, Scala D, Davis V, Wilson D, Stone M (1983). The short-term effects of three different strength-power training methods. Strength Cond J.

[CR31] Taaffe D, Pruitt L, Pyka G, Guido D, Marcus R (1996). Comparative effects of high and low intensity resistance training on thigh muscle strength, fiber area, and tissue composition in elderly women. Clin Physiol.

[CR32] Weiss LW, Coney HD, Clark FC (2000). Gross measures of exercise-induced muscular hypertrophy. J Orthop Sports Phys Ther.

[CR33] Wilborn CD, Taylor LW, Greenwood M, Kreider RB, Willoughby DS (2009). Effects of different intensities of resistance exercise on regulators of myogenesis. J Strength Cond Res.

